# Predicting Reading Fluency Growth from Grade 2 to Age 23 with Parental and Child Factors

**DOI:** 10.1080/10888438.2024.2346323

**Published:** 2024-04-27

**Authors:** Daria Khanolainen, Maria Psyridou, Kenneth Eklund, Tuija Aro, Minna Torppa

**Affiliations:** a Department of Teacher Education, University of Jyväskylä, Jyväskylä, Finland; b EDUCA flagship, University of Jyväskylä, Jyväskylä, Finland; c Department of Psychology, University of Jyväskylä, Jyväskylä, Finland; d Faculty of Education and Psychology, University of Jyväskylä, Jyväskylä, Finland; e Centre of Excellence in Learning Dynamics and Intervention Research (InterLearn), University of Jyväskylä, Jyväskylä, Finland

## Abstract

**Purpose:**

Reading fluency establishes the basis for the strong literacy skills needed for academic success. We aim to trace how reading fluency develops from childhood to adulthood and identify factors that influence this development.

**Method:**

In this study, 200 families were followed. All participating children (*N* = 200, 47% female) were ethnic Finns and spoke Finnish as their native language. The dataset included children’s reading fluency assessments (in Grades 2, 3, and 8 and at age 23), their self-reports, and parental questionnaires. For data analysis, growth curve models that included cognitive, motivational, and parental predictors were constructed.

**Results:**

Significant variations in both developmental rates and adult outcomes of reading fluency were found. The developmental rate was predicted by rapid automatized naming (RAN), letter knowledge, the formal home literacy environment (HLE) (measured in kindergarten) and reading motivation (measured in elementary school). Adult outcome (fluency at age 23) was predicted by RAN, letter knowledge, formal HLE, and parental dyslexia. Further, those who had parents with resolving reading difficulties were more likely to follow a resolving trajectory themselves compared with those whose parents had persistent reading difficulties.

**Conclusion:**

Our findings offer novel insights into how reading fluency develops into adulthood and identify key areas for future research to better understand the mechanisms behind reading fluency development.

Not all individuals achieve reading proficiency despite the targeted efforts and resources that many nations allocate to support reading development. Importantly, poor reading skills have enormous negative impacts on both individual lives and the global economy. Lower levels of reading skills are associated with lower life quality and mental health problems in adulthood (Aro et al., 2019; Eloranta et al., 2019; McLaughlin et al., 2014). A recent study estimated that the United States could be losing over two trillion dollars in annual income owing to low adult literacy rates (Rothwell, 2020).

Among the multiple components forming the core of reading ability, this study focuses on reading fluency and its development. In contexts with transparent orthographies, such as Finland, the country where the present study was conducted, reading dysfluency is a hallmark of reading difficulties (Aro & Wimmer, 2003). Moreover, reading fluency builds the foundation for developing reading comprehension, the ultimate goal of the reading process (Florit & Cain, 2011; Perfetti, 1985; Pikulski & Chard, 2005). Nevertheless, how reading fluency develops over the long run and what factors contribute to its faster development remain unclear. Exploring these factors could be beneficial for designing better support and intervention programs for individuals with reading difficulties.

The objectives of this study were twofold: to gain new insights into reading fluency development by analyzing its long-term trajectories from childhood to adulthood, and to identify which parental and/or child factors predict these trajectories (their intercepts and slopes). To this end, we used growth curve modeling and investigated the relationship between reading fluency trajectories from Grade 2 (age 8) to age 23 and several predictors (children’s cognitive skills, reading motivation, and task-avoidant behavior as well as parental education, history of dyslexia, income, and the home literacy environment [HLE]).

## Children’s cognitive skills as predictors of reading fluency

Considerable research has examined various early cognitive predictors of reading difficulties finding phonological processing skills to be a consistently strong predictor (for a systematic review, see Melby-Lervåg et al., 2012). They constitute the foundation for reading acquisition in alphabetical orthographies because reading requires association building between letters and sounds. However, the relationships between early phonological skills and later reading outcomes are stronger in more opaque orthographies (such as English) than in more transparent ones (Landerl et al., 2019; Ziegler et al., 2010). In the Finnish context, phonological skills have been found to be particularly predictive of reading accuracy and spelling, but not of reading fluency (Kairaluoma et al., 2013; Torppa et al., 2013).

Rapid automatized naming (RAN) is another cognitive skill that has long been considered key to fluent reading. Cross-linguistic research has repeatedly demonstrated that RAN is a unique predictor that has a strong direct effect on reading fluency development across different contexts and orthographies (Georgiou et al., 2016; Landerl et al., 2019). Norton and Wolf (2012, p. 429) argued that RAN is a good universal predictor because it is “a microcosm of the later developing, more elaborated reading circuit” and that RAN tasks and fluent reading share many of the same underlying mechanisms, “from eye saccades to working memory to the connecting of orthographic and phonological representations.” Protopapas et al. (2013) further argued that the brain handles RAN tasks and fluent reading similarly because they both require simultaneous processing of different information units incoming sequentially – as a person is articulating the current information unit, their brain is already processing the next one while anticipating yet another. The proposed mechanism has been substantiated by empirical evidence showing that RAN is more strongly related to serial word reading (words presented in texts or lists) compared with discrete/isolated word reading (Altani et al., 2020; Protopapas et al., 2013). Notably, in their study with Greek- and English-speaking children in Grades 1, 3, and 5, Altani et al. (2020) additionally found that discrete/isolated word reading development had already reached a plateau by Grade 3, while both serial word reading skills and RAN ability continued developing past that point (Altani et al., 2020). Other longitudinal research with older children indicates that RAN has strong long-lasting associations with fluency development at least until age 15 (e.g., Landerl & Wimmer, 2008; Lervåg & Hulme, 2009; Psyridou et al., 2023). For example, Landerl and Wimmer (2008) followed German-speaking children for eight years starting from Grade 1 and found that RAN measured at school entry continued to be a significant predictor of reading fluency until Grade 8. Studies conducted in the Finnish context have shown that a deficit in RAN specifically characterizes adolescents and adults with persistent and late-emerging reading difficulties (Eloranta et al., 2019; Torppa et al., 2015). Previous studies employing the same sample as the present study have also shown that RAN is one of the best predictors of reading fluency in childhood and early adolescence, but these studies did not include any fluency measures from adulthood (Eklund et al., 2018; Puolakanaho et al., 2007; Torppa et al., 2013).

The intelligence quotient (IQ) is another cognitive predictor possibly related to resolving difficulties. Although dyslexia can occur in people with any level of IQ (Ferrer et al., 2010; Tanaka et al., 2011), evidence suggests that high IQ can sometimes contribute to compensatory mechanisms that support reading development and help individuals with dyslexia mask and/or overcome reading difficulties (Moojen et al., 2020; Reis et al., 2000). Moojen et al. (2020) reported that higher verbal IQ levels, in particular, predicted more fluent reading in highly educated adults with dyslexia. Lefly and Pennington (1991), however, found that adults with resolved and persistent dyslexia did not differ in their full-scale IQ scores (scores combining verbal and non-verbal components). However, studies focusing on such exceptionality are rare and have very small samples. Additionally, a meta-analysis evaluating what predicts responses to reading interventions found that growth in reading fluency during interventions was not predicted by IQ (Scholin & Burns, 2012), although it is important to note that interventions are usually rather short and there is an obvious lack of research tracing long-term growth in reading fluency.

Letter knowledge, another early skill, is strongly predictive of reading development (Pennington & Lefly, 2001; Puolakanaho et al., 2007; Snowling et al., 2003). Using the same dataset as the present study, Torppa et al. (2010) showed that letter knowledge measured at age 5 was the best predictor of reading fluency at age 8, compared with all other early cognitive predictors (vocabulary, RAN, and phonological skills). Most recent Finnish research shows that letter knowledge not only strongly predicts early reading skills, but also predicts reading fluency in adolescence (Psyridou et al., 2023). However, whether letter knowledge can retain its predictive power in studies with adult-age outcomes is still unclear.

### Leisure reading and reading motivation as predictors of reading fluency

Understanding whether and how children’s own inclination to engage in reading activities can affect their developmental trajectories and educational outcomes represents an important research goal. Research suggests that reading skills are reciprocally associated with reading interest and the amount of leisure reading (Mol & Bus, 2011; Torppa et al., 2020; van Bergen et al., 2021). However, longitudinal studies spanning beyond early grades are scarce; thus, which (if any) of these factors has significant long-term influences on reading fluency development and whether the time of their presence (early childhood, adolescence, or adulthood) plays a role is unknown. In the Finnish context, Torppa et al. (2020) found in a longitudinal study that both reading fluency and comprehension were positively related to the amount of leisure reading, but the association with comprehension was stronger. Their longitudinal model, constructed from Grade 1 to Grade 9, revealed that better reading fluency and comprehension in early grades predicted more leisure reading, whereas more leisure reading contributed to better reading comprehension in later grades. van Bergen et al. (2021) employed the same sample as in our study, finding that earlier skills predicted more independent reading, which led to both stronger fluency and comprehension at later time points (in adolescence). Our study extends previous works by including a longer follow-up period and by examining whether these associations remain until adulthood.

In the context of school children’s development, task avoidance can significantly affect reading development. Task avoidance can be defined as the reluctance to engage in learning-related tasks requiring continuous effort and persistence. In 1986, Stanovich highlighted the possibility of early reading failure leading to snowballing consequences, as early negative experiences can make children more likely to be avoidant of learning situations, which further widens the skill gap between individuals with and without reading difficulties (Stanovich, 2009). Later empirical evidence indeed suggested a reciprocal relationship between task avoidance and reading skills (Eklund et al., 2013; Georgiou et al., 2017; Lundberg & Sterner, 2006). Most studies on the role of task avoidance in reading development, however, have been cross-sectional or limited to elementary school. In their longitudinal study tracing the stability of task avoidance from Grade 2 to age 20 in the same sample as in the current study, Syal and Torppa (2019) tested whether individuals with and without reading difficulties identified in Grade 2 differed in task avoidance levels at any time point. They found that Grade 2 children with reading difficulties were significantly more task-avoidant than their typically developing peers, but no significant difference at any other time point was observed. However, as the focus was on group comparisons of children with and without dyslexia, further investigation into whether task avoidance can predict children’s developmental rate in reading fluency is required.

### Parental characteristics and the home literacy environment as predictors of reading fluency

Various parental characteristics have been linked to children’s reading development. One is socio-economic status (SES), which is often measured through parental education level, parental income, or the combination of both. The association between SES measures and children’s skills may be attributed to parents with lower SES having insufficient resources to invest in their child’s development and/or having to spend most of their time working away from home (Bradley & Corwyn, 2002). A large-scale international study involving 43 countries found that SES predicted reading comprehension, but no relations with reading fluency were tested for (De Chiu & McBride Chang, 2006). More recently, another international study involving a dozen European countries revealed that children with lower SES engaged in fewer literacy activities at home prior to entering school and demonstrated lower reading comprehension skills in later elementary school compared with their peers from higher SES backgrounds (Hemmerechts et al., 2017). At the same time, studies examining the link between SES and reading fluency specifically have produced more equivocal findings (Inoue et al., 2018; Puglisi et al., 2017); thus, whether and what role parental SES plays in children’s reading fluency development remains unclear. It is important to note that even though SES has been found to be associated with different reading skills in all English-speaking countries, research has also shown that SES is linked with other parental and home-related factors, which are often omitted from research even though they could be more influential than SES (Buckingham et al., 2013).

In Finland, using the present sample, Aro et al. (2009) found that maternal education level was significantly related to reading fluency at age 8. Later, using the same sample, Torppa et al. (2015) reported that the sum score of maternal and paternal education did not differentiate between different groups of readers at age 15 (adolescents with typical reading, with resolving reading difficulties, with late-emerging difficulties, and with persistent difficulties). However, to date, longitudinal associations between parental education, income, and children’s adult-age outcomes have not been examined in the present sample.

Furthermore, multiple studies have found a significant positive association between the richness of the HLE and children’s literacy development (Bus et al., 1995; Flack et al., 2018; Grolig et al., 2019; Sénéchal & Lefevre, 2002). Literature often divides all literacy-related learning organized at home into two conceptually distinct types: formal and informal (Sénéchal & Lefevre, 2002). The formal HLE encompasses direct teaching activities aimed at literacy development, whereas the informal HLE encompasses meaning-related activities (shared reading and book-based discussions). The formal HLE reportedly contributes to the development of letter knowledge, early word recognition, and other emerging literacy skills; the informal HLE predicts language skills and reading comprehension (Manolitsis et al., 2011; Sénéchal & LeFevre, 2014). The dataset employed in the present study was explored by Torppa et al. (2006, 2007, 2022) in three studies examining the longitudinal influences of the HLE. In the first study, Torppa et al. (2006) investigated the development of letter knowledge and found that it was predicted by formal HLE. In the second study, Torppa et al. (2007) traced the development of various literacy-related skills and established that shared reading predicted vocabulary. Note that the follow-up in both studies stopped before school entry (at age 6.5). In the third study, which included a much longer follow-up, Torppa et al. (2022) found that the formal HLE (measured at age 4) predicted emerging literacy skills at age 5, whereas shared reading at age 8 predicted reading comprehension at age 15. However, in their analysis, they primarily focused on reading comprehension and included reading fluency from only one time point (age 8). Overall, HLE-related longitudinal research extending into adolescence and adulthood is currently lacking, and it remains unknown if any early learning activities organized at home before school entry can have long-term effects on reading fluency which are traceable later in life.

Finally, various studies have consistently found parental reading difficulties to be significant predictors of children’s reading ability across contexts (Esmaeeli et al., 2019; van Bergen et al., 2014), thus confirming that not only environmental but also genetic influences shape children’s developmental processes. Therefore, parental reading difficulties can act as a confounding factor in any study examining the role of family-related environmental characteristics in child development. For example, Aro et al. (2019) found that adults with a known history of learning difficulties are more likely to have lower education levels and face unemployment (diminishing their financial security). Thus, if parental reading skills are not considered as a potential confounding variable during analysis, then it remains unclear whether low SES itself influences children’s reading development or low SES stems from parents having learning difficulties that make their children genetically predisposed to the same difficulties and that this genetic predisposition is what actually explains children’s poorer reading skills (Buckingham et al., 2013).

Similarly, parental difficulties need to be considered in research on the HLE. Puglisi et al. (2017) reported that parental literacy teaching remained significantly predictive of children’s emerging reading skills, even after accounting for maternal language skills. However, they also found that shared reading stopped being predictive of children’s literacy skills once maternal language skills were considered. This suggests that the effect may be originating from maternal language skills, influencing both the amount of shared reading and children’s skills, rather than the amount of shared reading directly affecting children’s skills. Recent longitudinal research extending from age 2 to adolescence in the Finnish context indicated that early shared reading experiences can have long-lasting effects on reading development, even after controlling for parental reading skills (Khanolainen et al., 2020; Torppa et al., 2022). However, whether parental reading difficulties, SES, and/or early literacy-related activities organized at home can predict adult reading outcomes in offspring when all these predictors are added to the same model to avoid confounding remains to be tested. Moreover, to the best of our knowledge, ours is the first study to examine whether distinct developmental types of parental reading difficulties (compensated/resolving or persistent) can predict differential trajectories of children’s reading fluency development.

### The present study

Previous research has demonstrated that various factors potentially influence reading fluency trajectories, but longitudinal research reaching adult age is still needed to identify these factors. In view of this, the present study was guided by the following research questions: (1) What trajectories of reading fluency development do individuals exhibit over time (from age 8/Grade 2 to age 23)?, (2) What child factors (cognitive skills and motivation-related factors) predict individual differences in the trajectories of reading fluency development?, and (3) What parental factors (parental education, parental income, the HLE, and parental reading difficulties and their specific types) present when the child is in kindergarten or elementary school are associated with children’s reading fluency trajectories?

To answer these questions, we used the growth curve model, a well-known analytic approach for studying between-person differences in within-person patterns of change. Growth models allow the examination of the level/intercept and slope of development without using arbitrary cutoffs and are more flexible than traditional methods, as they require relatively fewer assumptions to be met (e.g., repeated measures analysis of variance). Thus, they provide more statistical power than more traditional quantitative methods (Curran et al., 2010). For example, they can be effectively applied to non-normal data (DeLucia & Pitts, 2006). Owing to their numerous advantages, growth curve models have long been used in educational research to produce valuable insights (Plewis, 2010).

Another important feature of our study is its long follow-up period. Most previous longitudinal studies followed children’s reading development only across the first few grades; very few studies with a follow-up until later grades of elementary school (e.g., Georgiou et al., 2012; Kim et al., 2012; Verhoeven & van Leeuwe, 2009) and until adolescence (e.g., K. Eklund et al., 2018; Francis et al., 1996; Georgiou et al., 2021; Landerl & Wimmer, 2008; Roman et al., 2009) exist. Importantly, none of the previous studies traced reading fluency development until adulthood.

Previous growth curve studies (Caravolas et al., 2013; Georgiou et al., 2021; Parrila et al., 2005; Peng et al., 2019; Verhoeven & van Leeuwe, 2009) revealed that in more consistent orthographies, the trajectory of reading fluency development follows a steep curve indicative of fast growth at the very start of reading instruction, after which the trajectory becomes much gentler. In less consistent orthographies, however, the initial growth is slower, and the changes along the trajectory are more gradual. Also, previous growth curve studies either included no predictors at all (Francis et al., 1996; Parrila et al., 2005; Verhoeven & van Leeuwe, 2009) or only cognitive predictors (Caravolas et al., 2013; Georgiou et al., 2021; Peng et al., 2019); thus, it remains unclear if any motivation-related and/or parental factors can predict individual differences in developmental rates and adult outcomes.

Against the background of existing evidence, we formulated the following hypothesis: Reading fluency will develop in the following way: between ages 8 and 9, there will be a period of most rapid growth, between ages 9 and 14, the growth rate will noticeably slow down, and between ages 14 and 23, growth will continue to decrease, possibly reaching a complete plateau. Due to the lack of previous growth curve studies conducted in consistent orthographies, we did not formulate a specific hypothesis regarding what predictors will be significant in the prediction of the slope and the intercept (set at the age of 23).

## Materials and methods

### Participants

Two hundred families expecting a child from 1993 to 1996 were recruited for the Jyväskylä Longitudinal Study of Dyslexia (JLD) and have been followed since the children were born (for details, see Lohvansuu et al., 2021). The present study included data from parents (parental reading skills were evaluated before the children were born, and parental questionnaires were collected when the children were aged 4, 6, 8, and 9 years) and children (children’s skills were assessed at age 5, in Grades 1, 2, 3, and 8, and at age 23; children’s self-reports were collected when they were in Grades 7 and 9 and 20 years old). Half of the participating children (*n* = 100 in Grade 2, *n* = 108 in Grade 3, *n* = 101 in Grade 8, and *n* = 75 at age 23) were at family risk (FR) for dyslexia owing to at least one parent having dyslexia, and the other half were age-matched controls (*n* = 78 in Grade 2, *n* = 92 in Grade 3, *n* = 81 in Grade 8, and *n* = 61 at age 23). The details of parental assessments can be found in Leinonen et al. (2001).

Participants in the FR group and controls were matched on parental education levels and parental IQ (all scores were above 80 on Raven’s Standard Progressive Matrices subtests B, C, and D [Raven et al., 1992]). All participating families spoke Finnish as a native language and were monolingual. Further, all children attended comprehensive (nonselective) public schools that followed the national curriculum.

### Children’s measures

#### Reading fluency

Separate sum scores for reading fluency were calculated at four time points (in Grades 2, 3, and 8 and at age 23). Word list reading, text reading, and pseudoword text reading scores were included in the total composite. Assessments using these tasks have been detailed by Eklund et al. (2015). Cronbach’s alpha reliability coefficients for the composites were high: .90 in Grade 2, .87 in Grades 3 and 8, and .86 at age 23. Because some task items were not identical at each time point (same instructions but different age-appropriate texts and word lists were offered), we counted the number of letters in the text reading task and divided this number by the seconds spent by each participant to read the text. Thus, the score at each time point reflected the number of attempted letters per second.

#### Cognitive skills

Children’s neurocognitive development was assessed using three measures: phonological awareness at ages 3 and 5, RAN at ages 5 and 6, and IQ at age 8 (in Grade 2).

#### Phonological awareness

At ages 4.5 and 5.5, participants’ phonological awareness was assessed using multiple measures, including segment identification, synthesis of phonological units, initial phoneme identification, and production (tasks from “Heps-Kups Land,” Puolakanaho et al., 2003; the phonological subtest of the Developmental Neuropsychological Assessment [NEPSY], Korkman et al., 1998). The two composite scores for phonological skills were combined into one variable. Cronbach’s alpha for the composite score was .74. All of these phonological tasks were previously described in detail (Aro et al., 2009; Torppa et al., 2022).

#### RAN

At ages 5.5 and 6.5, participants were given a standard RAN task: they were asked to name five objects graphically depicted. The objects were randomly repeated 6 and 10 times, respectively, and were presented in 10 × 5 and 6 × 5 matrices, respectively, at ages 5.5 and 6.5 (see Denckla & Rudel, 1976; Finnish version; Ahonen et al., 1999). The total completion time in seconds was recorded as a participant’s score for each matrix. Cronbach’s alpha for the composite score was .62.

#### IQ

A version of the Wechsler Intelligence Scale for Children III with nine subtests (Wechsler, 1991; Finnish version; Wechsler, 1999) was administered to assess children’s general intellectual functioning in Grade 2. This test version included five subtests from the verbal scale and four from the performance scale (the picture arrangement subtest was excluded). This test version was previously standardized with Finnish 8-year-old children and demonstrated a reliability of .76 (Wechsler, 1999).

#### Letter knowledge

At ages 5 and 5.5, participants were asked to name letters that were presented to them one at a time (each letter on its own page). The task included 23 uppercase letters. The Finnish alphabet has 29 letters, but six of them were excluded from the task because they occur only in words of foreign origin. The letters were arranged in four sets following the order of the Finnish alphabet, and participants received one point for each correct response (both the letter name and the corresponding phoneme were counted as correct responses). At age 5, all 23 letters were presented to each child. At age 5.5, however, testing was discontinued in cases where the child failed to name any letters in a set. Cronbach’s alpha for the composite score was .94.

#### Task avoidance

Children’s task avoidance in Grade 2 was evaluated by their mothers, who answered five questions from the Behavioural Strategy Rating Scale (Eklund et al., 2013; Onatsu-Arvilommi et al., 2002). This short measure included the following items: 1. When dealing with a challenge, does the child tend to switch to doing something else instead of persevering?, 2. Does the child willingly engage even with the most difficult tasks? (item was reversed), and 3. Does it seem that the child easily gives up on tasks?, 4. Is the child persistent when dealing with tasks? (item was reversed), and 5. When encountering difficulties while dealing with a task, does the child switch his/her attention to other things? Cronbach’s alpha for the composite score was .89. Children’s task avoidance in Grades 7 and 9 was measured with the same five items from the Behavioural Strategy Rating Scale but revised to be a self-report measure. Cronbach’s alpha for the composite score was .83.

#### Reading motivation and leisure reading

Participants’ reading motivation in Grades 2 and 3 was reported by mothers using a questionnaire with a 5-point Likert scale: 1. How often does your child independently read books or magazines (1 = never to 5 = many times per day)?, 2. What is the typical duration of your child’s independent reading episode (1 = 5 min to 5 = more than 45 min)?, and 3. How interested is your child in book reading (1 = not at all interested to 5 = very interested)? The total score for reading motivation was calculated as the mean of the six items (three items per measurement point). Cronbach’s alpha reliability coefficient was .85.

Leisure reading in Grades 7 and 9 was assessed with both participants’ self-reports and parental reports. The two self-report items included one question asked at each time point (“How much do you read in your free time?”). Parental reports were completed by mothers and included one question asked at each time point (“How often does your child read books in their free time?”). All four items were answered on the same 5-point Likert scale (1 = every day, 2 = 4–6 times a week, 3 = 1–3 times a week, 4 = less than 1–3 times a week, and 5 = never). Cronbach’s alpha for the four items was .83. The original leisure reading items were then reversed to match the scale of reading motivation so that higher scores indicated more leisure reading.

### Parental measures

#### Parental education

Before the birth of the participating children, both their mothers and fathers were screened and asked to report information on their general, vocational, and tertiary education. This information was used to create two parental education variables on a 7-point Likert scale ranging from the lowest duration of education to the longest, that is, from 1 (only general/no post-compulsory education) to 7 (post-graduate university degree). For each participating child, a composite score of their father and mother’s education levels was calculated.

#### Parental income

When children were in Grade 3, both mothers and fathers were asked to report their individual monthly income using a 14-point scale ranging from 1 (less than 300 euros) to 14 (over 4800 euros). For each participating child, a composite score of their father’s and mother’s incomes was calculated.

#### Parental reading difficulties

To identify parental reading difficulties, clinical interviews and cognitive and reading assessments were used. Participants were considered to be parents without reading difficulties (i.e., controls) if their scores did not fall lower than one standard deviation below the mean in all assessments and they reported having no reading difficulties in their family. Participants were identified as parents with reading difficulties if they reported reading difficulties for themselves and for at least one close relative and scored below at least one standard deviation in cognitive and/or reading assessments (for full details, see Leinonen et al., 2001). Based on this information, a binary variable was created (0 = control participants and 1 = participants who have at least one parent with difficulties). All parents with reading difficulties were further divided into those with persistent and those with resolved difficulties. Parents who self-reported childhood reading difficulties in a clinical interview and demonstrated poor cognitive skills but scored higher than one standard deviation below average on reading and spelling tests were identified as parents with resolved reading difficulties, whereas parents who not only self-reported childhood difficulties and demonstrated poor cognitive skills but also demonstrated poor reading skills were identified as parents with persistent difficulties. One binary variable was created for the control group (0 = participants who had one parent with resolved difficulties, and 1 = participants who had one parent with persistent difficulties).

#### HLE

The HLE was assessed with a parental questionnaire that included items about shared reading practices and literacy-related teaching. The questionnaire with items focusing on shared reading was administered to families with preschool children (aged 4, 5, and 6 years). It asked how frequently the mother read to the child, how frequently the father read to the child, how frequently stories were read to the child at home, how long a typical shared reading episode lasted, and what was the total amount of time in a day adults spent on reading together with the child. All items were answered on a 5-point scale. The total composite score for shared reading included all items from the three time points and had a Cronbach’s alpha of .87.

Four items about at-home teaching were added to the HLE questionnaire administered when the children were 4.5 years old. These items ask about how frequently letter names were taught to the child, phonemes were taught to the child, the child was taught to blend letters, and the child was taught to blend phonemes. These items were answered on a 5-point Likert scale ranging from 1 (not at all) to 5 (every day). An additional alternative, 6 (not anymore because the child is skillful already), was also offered. This alternative created a problem when the sum score for the formal HLE was calculated because it referred to teaching in the past rather than in the present. The number of people who selected 6 was rather small; thus, all values of 6 were recoded as 5. Next, a composite variable for literacy teaching was created by calculating the average of the responses to the four questions. Cronbach’s alpha was .78.

### Statistical analysis

To investigate how reading fluency develops from Grade 2 to age 23, we constructed growth curve models (Grade 2, *n* = 178; Grade 3, *n* = 200; Grade 8, *n* = 182; age 23, *n* = 136). This analysis was conducted with Mplus 7.4 (Muthén & Muthén, 1998–2012), and all models were estimated using maximum likelihood with robust standard errors.

Retention of study participants over the years was generally good (89% of the entire sample participated in assessments in Grade 2, 100% in Grade 3, 91% in Grade 8, and 68% at age 23). At the last time point, when the number of participants was the lowest, 136 participants remained. For a growth model, recruiting at least 100 participants is recommended (Curran et al., 2010). Little’s missing completely at random test indicated that all variables were missing completely at random (χ^2^ (2398) = 2489.590, *p* = .09). In addition, all variables that had missingness over 20% were more closely examined for possible systematic missingness, but no specific patterns were identified. More information on this is presented in the Appendix. Missing data were handled using full information maximum likelihood (FIML) estimation, which is less likely to produce biased results than other common techniques, because it utilizes all information from all participants. FIML has long been regarded as one of the best ways to handle missingness in general and when using growth curve models in particular (Mueller & Hancock, 2019). No extreme outliers were detected in the selected reading measures, and no participants were excluded from the analysis.

For the baseline growth model, the loadings on the intercept were fixed to 1, the loading on the slope at the first time point (reading fluency in Grade 2) was fixed to − 1, the loading at the last time point (reading fluency at age 23) was fixed to 0, and the loadings for the second and third time points (Grades 3 and 8, respectively) were allowed to be freely estimated. In this model, the slope indicates the developmental rate from Grade 2 to age 23, whereas the intercept indicates the reading fluency outcome at age 23. The slope and intercept were allowed to covary. This parameterization was selected to focus on adult-age skill prediction, which is an obvious knowledge gap, as most previous studies focus on the prediction of reading skills in childhood (and rarely in adolescence).

**Figure 1.**
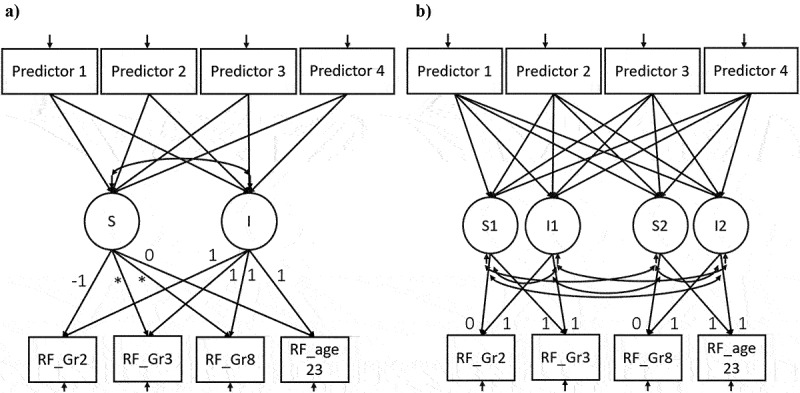
a) the specification of the main growth curve models 1–5 for reading fluency and the predictors: b) the specification of the additional models 1–5 with two separate growth curves.

*Note*. Models 1, 2, 3, 4, and 5 were very similar and only differed on their predictors. The exact list of predictors included in each of the four models can be found in Table 3. Asterisks (*) indicate that the loadings were allowed to be estimated freely.

Once the optimal baseline growth curve model was established, it was expanded to include the predictors (Figure 1a). Model 1 included all children’s cognitive skills and letter knowledge as predictors, whereas Model 2 included children’s motivation-related and socio-emotional scores. Model 3 included parental variables and the HLE. After all significant predictors were identified with Models 1, 2, and 3, Model 4 was constructed and included only significant predictors from each model.

Considering that we were working with a prospective FR sample (half of the children in the sample had at least one parent with dyslexia), we tested if FR was a moderator by additionally running Models 1, 2, 3, and 4 as multi-group models. To this end, we performed chi-square difference tests to compare unconstrained and constrained models (models where paths are constrained to be equal across groups). No significant group differences were observed in any of the estimates between the FR group and the controls (test statistics provided in Table 3), indicating that parental difficulties did not moderate any of the found associations. Therefore, below, we report the results obtained from the full sample models only.

Furthermore, to examine the impact of FR type on children’s reading fluency development, we ran Model 5: a model with two dummy variables as predictors indicating if parental difficulties were persistent or resolving. Controls (those whose parents had no dyslexia) were the reference group in this analaysis.

Finally, each of these five growth curve models were transformed into an additional model with two separate growth specifications (this type of model is shown in Figure 1b). These additional models allowed us to test if the change in the earlier growth rate (from age 8 to 9) was predictive of the later growth rate (from age 14 to 23). However, these additional models did not provide insight into what happened at the interval between ages 8 and 14. For this reason the findings from these models play a secondary role in this article.

## Results

**Figure 2.**
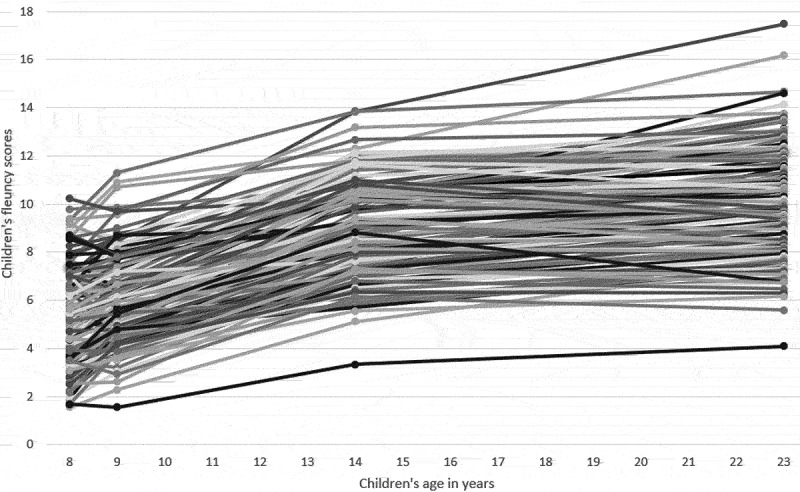
All reading fluency trajectories.

Table 1 presents the descriptive statistics for all variables, and Table 2 shows the correlations among all variables. Figure 2 illustrates the individual growth curves for all participants in the sample and the general developmental trend, showing that reading fluency develops throughout the study, although its developmental pace gradually decreases. Except for children’s RAN, all variables exhibited a normal distribution, with skewness and kurtosis values falling within the acceptable range. RAN however demonstrated a rather high kurtosis value of 5.44, primarily due to the presence of three outliers (they had scores greater than three standard deviations above or below the sample mean). To address this issue, we ran the model inclusive of RAN twice: once using the original dataset and once using the dataset with winsorized outliers (the kurtosis value for RAN in this dataset decreased to 1.47, while the skewness value changed from 1.76 to 1.02). The two models showed consistent results. For this reason, only the results obtained using the original RAN scores are presented below.

**Table 1.** Descriptive statistics for all variables.

	N	Minimum	Maximum	Mean	SD	Skewness (Std. Error)	Kurtosis (Std. Error)
**Reading fluency**							
Grade 2	178	1.23	10.22	5.15	2.06	.25 (.18)	−.70 (.36)
Grade 3	200	1.55	11.30	5.83	1.87	.38 (.17)	−.20 (.34)
Grade 8	182	2.96	13.85	8.69	2.01	−.08 (.18)	−.10 (.36)
Age 23	136	4.10	17.48	10.18	2.21	.22 (.21)	.37 (.41)
**Children’s phonological awareness**							
	159	−2.18	1.58	−.18	.72	−.07 (.19)	−.03 (.38)
**Children’s RAN**							
	195	31.50	144.35	59.91	16.57	1.76 (.17)	5.44 (.35)
**Children’s Verbal IQ**							
	198	72	137	99.84	11.60	.25 (.17)	−.19 (.34)
**Children’s Performance IQ**							
	198	64	135	100.83	13.20	−.09 (.17)	−.29 (.34)
**Children’s letter knowledge**							
	197	.00	23.00	11.59	7.44	−.00 (.17)	−1.43 (.34)
**Reading motivation (ages 8–9)**							
	163	1.50	3.67	2.78	.57	−.31 (.19)	−.53 (.38)
**Task avoidance (age 8)**							
	173	.00	3.60	1.52	.80	.25 (18)	−.29 (.37)
**Task avoidance (ages 13–15)**							
	133	1.80	4.70	3.20	.66	.11 (.21)	−.54 (.42)
**Leisure reading (ages 13–15)**							
	112	1.00	5.00	3.36	.92	−.60 (.23)	−.21 (.45)
**Parental education**							
	198	1.00	7.00	4.03	1.09	.66 (.17)	.77 (.34)
**Parental income**							
	150	1.50	14.00	7.48	1.96	.01 (.20)	.96 (.39)
**HLE (shared reading)**							
	156	1.00	4.53	2.89	.58	.09 (.19)	.64 (.39)
**HLE (teaching letters)**							
	190	1.00	5.00	2.26	.91	.89 (.18)	.49 (.35)

**Table 2.** Pearson correlation coefficients between all variables.

Variable, age in years	1	2	3	4	5	6	7	8	9	10	11	12	13	14	15	16	17	18
1. Reading fluency, 8	1																	
2. Reading fluency, 9	.89***	1																
3. Reading fluency, 14	.74***	.79***	1															
4. Reading fluency, 23	.72***	.76***	.81***	1														
5. Parental education	.17*	.09	.11	.13	1													
6. Parental income	.07	.00	.04	.01	.43**	1												
7. Parental dyslexia, y/n	−.33***	−.24***	−.27***	−.25***	−.10	−.04	1											
8. HLE, shared read 4–6	.08	.06	−.04	.07	.09	−.04	.06	1										
9. HLE, teaching, 4	.21**	.14	.18*	.35***	.09	−.01	−.18*	.13	1									
10. Verbal IQ, 8	.37***	.34***	.23 **	.35***	.17*	−.00	−.12	.08	.19*	1								
11. Perform. IQ, 8	.27***	.21**	.15*	.17*	.14	.09	−.08	.15	.07	.46***	1							
12. RAN, 5–6	−.38***	−.39***	−.43***	−.39***	−.13	.00	.24***	.07	−.07	−.31***	−.13	1						
13. Phonol. skills, 3–5	.36***	.31***	.25***	.34***	.25***	.17*	−.31***	.15	.33**	.48***	.31***	−.36***	1					
14. Letter know., 5	.55***	.47***	.38***	.34***	.22**	.16*	−.21**	.14	.34***	.47***	.26***	−.43***	.67***	1				
15. Reading motiv., 8–9	.49***	.42***	.21**	.27**	.08	−.02	−.07	.34***	.22**	.33***	.32***	−.08	.31***	.28***	1			
16. Task avoidance, 8	−.16*	−.23**	−.18*	−.16	−.05	−.07	.11	−.23**	.00	−.20**	−.16*	.13	−.17*	−.18*	−.25**	1		
17. Task avoid., 13–15	−.01	−.07	−.11	−.08	−.21*	−.13	.09	−.11	−.20*	−.11	−.07	.07	−.03	−.04	−.06	.19*	1	
18. Leisure read., 13–14	.06	.12	.07	.14	.07	−.06	.01	.22*	.03	.09	.14	−.01	.15	.07	.36***	−.17	.20*	1

*p < .05, **p < .01, ***p < .001.

The baseline growth curve model without any predictors demonstrated an excellent fit to the data (χ^2^ (3) = 4.08, *p* = .25, CFI = .99, TLI = .99, RMSEA = .06, 90% CI [.00, .19], SRMR = .04). The slope and intercept of the baseline model were positively correlated (.40, *p* < .001), suggesting that faster developmental rates were predictive of better reading fluency outcomes at age 23. The means and variances of the slope and intercept were all statistically significant (*p* < .001), pointing to the significant differences among participants’ developmental rates and adult outcomes. These differences can also be seen in Figure 2. The baseline model additionally revealed that the biggest change in skills occurred between ages 9 and 14 (the change in slope between ages 8 and 9 was .15, between ages 9 and 14 it was .57, and between ages 14 and 23 it was .28).

**Figure 3.**
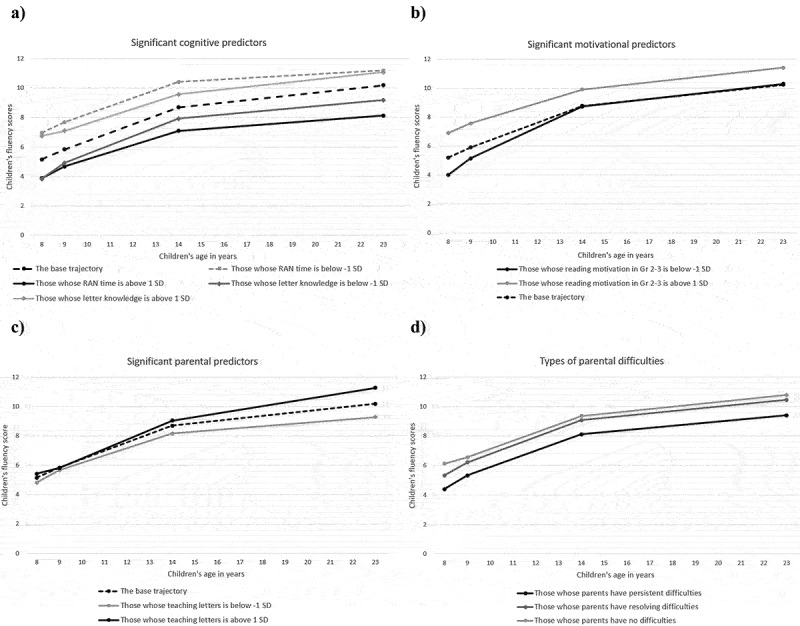
The associations between significant predictors and the reading fluency trajectory.

*Note*. In Figures a-c, the base trajectory was constructed using the mean scores of reading fluency derived from the whole sample. The other trajectories were constructed using the mean scores of reading fluency derived from the subsamples of those who demonstrated a) RAN, b) letter knowledge, c) reading motivation above or below one standard deviation in the total sample, or d) those who received the amount of literacy-related teaching that was above or below one standard deviation in the total sample.

The fit statistics indicated an acceptable fit for all models with predictors (Table 3). Table 3 lists all *R*^2^ values for intercepts and slopes. In Model 1, RAN and letter knowledge were the only significant predictors. RAN was negatively associated with both the intercept and slope, suggesting that longer time spent completing RAN tasks at ages 5 and 6 predicted both slower reading development and lower reading outcomes at age 23 (Figure 3a). Letter knowledge demonstrated at age 5 negatively predicted only the slope. As observed in Figure 3a, those with stronger letter knowledge showed better reading skills in the beginning, but their developmental curve later became gentler.

**Table 3.** The fully standardized estimates of the models and the model fit statistics.

	Intercept	Slope	Model fit statistics and Chi-square difference tests
	(age 23 level) Estimate (SE)	Estimate (SE)	
**Model 1: Cognitive predictors**			
Verbal IQ, age 8	.118 (.100)	.010 (.144)	Model 1 fit: x2 (13) = 25.83, *p* = .02, CFI = .98, TLI = .96, RMSEA = .07 (90% CI.03–.11), SRMR = .04. Chi-square difference test to compare if FR and control models are different: Δχ2(10) = 16.17, *p* = .095
Performance IQ, age 8	.013 (.077)	−.087 (.130)	
RAN, ages 5–6	−.374 (.075)***	−.316** (.108)	
Phonological awareness, age 5	−.073 (.109)	−.014 (.181)	
Letter knowledge, age 5	.200* (.094)	−.339* (.169)	
R-square	.274 (.059)***	.141 (.069)*	
**Model 2: Motivational predictors**			
Reading motivation, ages 8–9	.184 (.101)	−.395 (.123)**	Model 2 fit: x2 (11) = 18.88, *p* = .06, CFI = .99, TLI = .98, RMSEA = .06 (90% CI.00–.10), SRMR = .04. Chi-square difference test to compare if FR and control models are different: Δχ2(21) = 28.21, *p* = .134
Task avoidance, age 9	−.130 (.089)	−.035 (.108)	
Task avoidance, ages 13–15	−.135 (.095)	−.197 (.126)	
Leisure reading, ages 13–15	−.020 (.097)	.039 (.137)	
R-square	.084 (.047)	.181 (.096)	
**Model 3: Parental predictors**			
Parental education	.114 (.091)	.017 (.112)	Model 3 fit: x2 (17) = 29.54, *p* = .03, CFI = .98, TLI = .97, RMSEA = .06 (90% CI.02–.09), SRMR = .04 Chi-square difference test to compare if FR and control models are different: Δχ2(8) = 9.99, *p* = .266 (we compared the models in which parental difficulties were excluded from predictors because the family risk variable was used for grouping)
Parental income	−.058 (.124)	−.025 (.135)	
Parental dyslexia	−.208 (.076)**	.047 (.105)	
Shared reading, ages 4–6	−.045 (.570)	−.169 (.118)	
Teaching letters, 4	.217 (.076)**	.226 (.103)*	
R-square	.119 (.048)*	.069 (.054)	
**Model 4: All significant predictors**			
Parental dyslexia	−.116 (.077)	.044 (.097)	Model 4 fit: x2 (16) = 30.17, *p* = .02, CFI = .98, TLI = .97, RMSEA = .06 (90% CI.02–.10), SRMR = .04.
RAN, ages 5–6	−.384 (.072)***	−.288 (.109)**	
Letter knowledge, age 5	.103 (.083)	−.371 (.116)**	
Reading motivation, ages 8–9	.142 (.086)	−.375 (.104)***	
Teaching letters, age 4	.122 (.076)	.315 (.110)**	
R-square	.291 (.056)***	.323 (.098)**	
**Models 5: Types of parental dyslexia predictors (dummy predictors; the controls are the reference group)**			
Persistent parental difficulties	−.313 (.073)***	−.059 (.105)	Model 5 fit: x2 (7) = 10.91, *p* = .14, CFI = .99, TLI = .99, RMSEA = .05 (90% CI.00–.11), SRMR = .05
Resolving parental difficulties	−.038 (.099)	.137 (.118)	
R-square	.092* (.043)	.027 (.034)	

*p < .05, **p < .01, ***p < .001.

In Model 2, reading motivation in Grades 2–3 was a negative predictor of the slope. This indicates that those with lower levels of reading motivation at the beginning of the learning to read process in Grades 2–3 demonstrated steeper developmental slopes. Figure 3b illustrates this finding: those who had lower levels of reading motivation at ages 8 and 9 had lower reading fluency at the beginning of their learning trajectories but faster rates of reading development. More rapid development allowed them to gradually reach the same level of reading fluency as those who demonstrated average levels of reading motivation in elementary school. At the same time, those who had a higher motivation to read consistently demonstrated higher levels of reading fluency at all time points compared with those demonstrating average and lower levels of motivation. Assessments of later leisure reading and task avoidance did not predict either the intercept or the slope.

In Model 3, with parental predictors, parental reading difficulties negatively predicted the intercept, whereas literacy-related teaching positively predicted both the intercept and slope. This indicates that the participants who had parents with reading difficulties were more likely to demonstrate lower levels of reading fluency at age 23 than the participants without such a family history. The fact that the slope was not significantly predicted suggested that the reading trajectories of those with and without parental difficulties were mostly parallel until age 23. Moreover, this model and Figure 3c suggest that those who received more at-home teaching developed reading fluency at a faster rate and achieved higher outcomes at age 23 than those who did not receive such training.

All significant predictors identified with Models 1, 2, and 3 were added as predictors in Model 4. This model shows the unique contribution of each predictor when all other significant predictors are present. The analysis revealed that all included predictors, except for parental reading difficulties, were once again significant. Further, similar to Model 1, Model 4 revealed that RAN was a significant predictor of both the intercept and slope, indicating that stronger RAN contributes to faster reading development and better reading fluency in adulthood. In line with Models 1 and 2, Model 4 showed that letter knowledge and reading motivation were both negatively associated with the slope but had no relation with the intercept, showing that participants with higher letter knowledge and reading motivation followed gentler developmental trajectories. Finally, unlike Model 3, Model 4 indicated that literacy-related teaching was predictive of only the slope, suggesting that more literacy-related teaching was associated with faster reading development.

An analysis with dummy variables showed that the type of parental difficulty can be an important predictor. Model 5 demonstrated that having a parent with persistent dyslexia significantly predicted poor reading fluency at age 23, whereas having a parent with resolved reading difficulties was not significantly predictive of either the slope or the adult-age outcome. Figure 3d illustrates that those who had one parent with resolved difficulties had lower reading fluency in Grade 2 than the controls, but higher reading fluency than those who had one parent with persistent difficulties. By Grade 3, those with resolved parental dyslexia reached almost the same reading fluency level as the controls. Further, those with persistent parental dyslexia consistently demonstrated lower reading fluency at all time points, but their overall trajectory was parallel to the trajectory of those with resolved parental dyslexia.

The results from these main models were consistent with the results from the additional models that included two separate growth curves. Notably, the change in the first growth curve (between ages 8 and 9) did not predict the change in the second growth curve (between ages 14 and 23). A more detailed overview of these additional results is available in Appendix.

## Discussion

In this study, we constructed a growth curve model of reading fluency development spanning 15 years (from age 8 to age 23). In line with previous growth curve research conducted in contexts with consistent orthographies (Caravolas et al., 2013; Georgiou et al., 2021; Parrila et al., 2005; Verhoeven & van Leeuwe, 2009), our study revealed that in early grades, Finnish children follow a spurt of reading fluency development after which they slow down. It is important to note, however, that the trajectories in our study started only at age 8 (Grade 2), and for this reason, we missed the period of most rapid development during Grade 1. At the same time, our study had an exceptionally long follow-up, allowing us to further existing research by demonstrating that reading fluency continues developing past adolescence into adulthood even though the rate of development decreases over time.

Moreover, our growth curve model elucidates significant variation in not only the intercepts (adult outcomes) but also the slopes (developmental rates), indicating that the individual rank order in reading fluency is not fixed and can in fact gradually change. Previous research with the present sample showed that some learners with reading difficulties identified in Grade 2 (age 8) followed a resolving trajectory in their fluency development; thus, their reading difficulties were not confirmed in Grade 8 (age 14) (Torppa et al., 2015). In our study, when examining individual reading fluency trajectories (Figure 2), we saw that some learners continued improving their skills past age 14, while others remained on the same skill level or even regressed from ages 14 to 23. In addition, patterns observed in Figure 3(a,b) indicate that the majority of significant predictors (RAN, letter knowledge, reading motivation) affected the shape of the slope only in the beginning. Past age 14, the lines constructed for different levels of predictors remained mostly parallel, indicating that none of these predictors contributed to the change in the rank order improvement past age 14. Overall, this means that even though reading fluency development with potential rank order improvement is possible for some individuals after Grade 8 and during young adulthood, it remains largely unclear what factors drive this improvement. Future research should continue to explore the role of different predictors, including those measured during and past secondary school.

Our results further showed that faster RAN measured when children were in kindergarten predicted faster reading development and stronger adult reading fluency. Earlier longitudinal studies (Landerl & Wimmer, 2008; Lervåg & Hulme, 2009), including the studies conducted with the same sample as that of the present study (Eklund et al., 2018; Puolakanaho et al., 2007; Torppa et al., 2013), have already identified RAN as one of the best predictors of reading fluency and of resolving reading difficulties among school-age children and adolescents. In addition, Eloranta et al. (2019) found in a clinical sample that childhood RAN was the only predictor that differentiated adults with resolving reading difficulties from those with persistent difficulties. Our study extended these findings using a non-clinical sample and a different statistical analysis approach with a mostly different set of predictors.

Another significant predictor identified in our analysis was letter knowledge. Unlike RAN, however, it predicted the slope of reading development but not the adult outcome. This finding aligns with earlier research that traced reading fluency until adolescence (Eklund et al., 2018; Psyridou et al., 2023), showing that preschool RAN was a stronger predictor of reading fluency at age 14 compared with preschool letter knowledge. Conversely, previous studies with younger children showed that letter knowledge played a more important role than RAN in predicting reading fluency during the early grades at the stage when children’s reading was not yet fully automatized (Torppa et al., 2010). Together with earlier evidence, our study suggests that RAN is a particularly important predictor of reading skills in readers who have already reached the stage of automatic word recognition. This is likely because both fluent reading and RAN essentially entail concurrent processing of different information units presented in a specific sequence (Altani et al., 2020). It is also important to note here that although preschool letter knowledge did not predict reading fluency at age 23, there was a significant correlation between these two variables (Table 2). The likely reason for this seeming inconsistency may be attributed to the presence of other predictors in the growth curve model that were related to letter knowledge and demonstrated stronger predictive power for the outcome at age 23.

Children’s reading motivation in Grades 2 and 3 was another significant predictor of reading outcomes at age 23. This finding corroborates earlier research showing an association between reading fluency and motivational factors (Eklund et al., 2013; Georgiou et al., 2017). An important additional insight that our study provides is that higher levels of reading motivation did not predict a steeper developmental slope, suggesting that this characteristic does not contribute to resolving reading difficulties. Indeed, the visual representation of how reading motivation was related to the reading fluency trajectory (Figure 3b) indicated that children with high levels of motivation started following differentiated trajectories already in Grade 2 and did not gain additional advantage over time.

Evidence suggesting that improving reading motivation in early grades leads to better reading fluency in adulthood is limited; nevertheless, fostering reading motivation in children, particularly intrinsic motivation, is crucial because it has a reciprocal relationship with reading comprehension development (Hebbecker et al., 2019; Schiefele et al., 2016). Moreover, note that, in our study, reading motivation was not repeatedly measured across a long period; thus, it is unclear whether reading motivation sustained at a high level over an extended time period could be a factor contributing to resolving difficulties. Further research is required to address this knowledge gap and test whether reading motivation has cumulative effects on reading fluency development, especially considering that in our study, learners who initially demonstrated low reading motivation later reached the same level of reading fluency as those who had demonstrated average reading motivation. This development may be attributable to their initially low levels of reading motivation improving over time, thus helping their reading fluency to also improve. However, the reverse is also possible: those whose initial reading difficulties resolved with time may have gradually started demonstrating higher levels of reading motivation. Previous research suggests that the association between reading skills and reading motivation seems to be reciprocal and potentially runs more strongly from skills to motivation than from motivation to skills (van Bergen, Hart, et al., 2021). For the related construct of leisure reading, we found no significant effects; this finding is in line with a previous study that used another Finnish sample (Torppa et al., 2020).

We also found that parental reading difficulties were a significant negative predictor of reading fluency; this finding is consistent with most previous research (Esmaeeli et al., 2019; Snowling & Melby-Lervåg, 2016; van Bergen et al., 2014). However, our study contributes to this literature by showing that the influence of parental reading difficulties goes beyond childhood and adolescence and that it is also negatively predictive of adult reading outcomes. At the same time, parental reading difficulties stopped being significantly predictive in the final model with all significant predictors most likely because they share the same variance with children’s RAN.

Another novel insight provided by our study is that the specific type of parental reading difficulties was predictive of children’s reading fluency development rates: those who had parents with resolving difficulties were more likely to follow a resolving trajectory themselves, whereas those who had parents with persistent difficulties were more likely to also demonstrate persistent difficulties. Visual representations of the trajectories (Figure 3d) indicated that most of the improvement in the resolving group occurred by age 9. After this time point, groups with different types of parental reading difficulties followed parallel trajectories. Further research is needed to establish whether reading difficulties resolve over time owing to a specific genetic predisposition or because of the influence of certain environmental factors not included in this study.

Finally, in line with previous research (Manolitsis et al., 2011; Sénéchal & LeFevre, 2014), we found that the formal HLE (literacy-related teaching organized at home) provided at age 4 was positively associated with children’s reading fluency. Most previous research, however, only included early word recognition abilities and emerging reading skills measured in the early grades. Our study extends the existing literature by including skills from not only adolescence but also adulthood. The inclusion of data from later time points enabled us to test whether the HLE had long-lasting associations with reading development. Torppa et al. (2022) previously demonstrated that the informal HLE (shared reading) had lingering effects on reading comprehension. The researchers were able to predict children’s skills at age 15. Our study adds to this finding by showing that the formal HLE could be an influential factor in the long-term development of reading fluency, as at-home literacy teaching organized prior to school entry predicted differentiated developmental trajectories of reading fluency. These trajectories were at very similar levels in early grades, but gradually diverged, reaching the maximum gap at age 23. In fact, at-home literacy teaching organized at age 4 turned out to be the only significant predictor that affected the slope of trajectories past age 14. This pattern, however, makes us pause before recommending that parents engage in more at-home teaching if their goal is to facilitate faster reading fluency development. We cannot exclude the possibility that the association between at-home teaching and skill growth might have emerged owing to the influence of an unknown confounding variable that is currently missing from our analysis. Parental skills were controlled for; thus, another important factor (perhaps children’s early interest in learning letter names potentially reflected their higher enjoyment of learning activities in general, children’s mastery orientation, or factors related to certain skills, practices, or beliefs shared by family members, none of which we assessed in this study) might have caused this association.

All other included predictors were found to be insignificant (IQ, phonological awareness, task avoidance, leisure reading, parental education, parental income, and shared reading). However, our findings do not preclude possible associations between these predictors and reading fluency at certain time points. In fact, the table with Pearson’s correlations (Table 2) revealed that IQ, phonological awareness, task avoidance, and parental education were significantly related to some or even all reading fluency variables, suggesting that these predictors most likely lost their significance in the growth curve model because they overlapped in the variance they explained with other stronger predictors. Pearson’s correlations additionally indicated that reading fluency was not related to leisure reading, parental income, and the informal HLE (shared reading), possibly because these predictors do not significantly affect how reading fluency develops in the Finnish context. Previous longitudinal research showed that the amount of independent leisure reading (Torppa et al., 2020; van Bergen, Vasalampi, et al., 2021) and shared reading with parents (Torppa et al., 2022) contributed to improvements in reading comprehension in particular but not in reading fluency.

### Limitations

Our results should be interpreted with certain caveats in mind. First, longitudinal confirmatory factor analysis (CFA) revealed that there was no factorial invariance of model parameters across all four time points included in our growth curve model, though there was factorial invariance for the first two time points and for the last two time points. Yet, we decided it was acceptable to use fluency scores from all four time points in the same growth model because 1) high Cronbach’s alphas reported for each time point (.90 in Grade 2, .87 in Grades 3 and 8, and .86 at age 23) suggested the unidimensionality of our fluency measures and 2) reading fluency demonstrated high longitudinal stability (see Table 2 for high correlations between the fluency scores measured at different times). In view of CFA showing factorial invariance across the first two time points, even though the fluency tasks in Grades 2 and 3 were different, we believe that factorial non-invariance detected across the four time points did not arise due to the fluency tasks being slightly different. Rather, this non-invariance most probably stemmed from the fact that somewhere between the second and third time points (between ages 8 and 14), the mechanisms behind the reading process change as children transition from one developmental stage to another.

Second, our sample was not randomly selected, as half of the participants had parents with dyslexia. Thus, our results cannot be fully applied to a general population. However, we ran additional multi-group models to test if familial history acted as a moderating factor and established that no differences existed between the FR and control groups. Nevertheless, it is important to highlight that it was previously found in a general population sample that the average adult reading speed in Finnish was 161 words per min ( = 17.98 letters per second) (Trauzettel-Klosinski et al., 2012). In our study, however, at the adult age, the reading speed was 10.18 letters per second. The main two reasons that likely contributed to the decreased average speed in our sample are 1) the high number of participants at FR for dyslexia elevated the number of those with poor reading fluency in adult age and 2) the inclusion of the pseudoword task in the assessment batteries (in contrast, Trauzettel-Klosinski et al. only used real words in their assessments).

Note also that the Finnish context is unique in many ways. For example, the Finnish language has one of the most consistent orthographies in the world, Finnish children start school at age 7, special education is widely accessible, and access does not require a dyslexia diagnosis. These contextual characteristics further reduced the generalizability of our results. In view of these limitations, we recommend that future studies test similar growth curve models in other contexts using larger and more representative samples and including a wider range of predictors.

Third, the predictors’ data were collected using either parental reports or participants’ self-reports, which are subject to social desirability bias; nevertheless, the concepts used in our study as predictors are commonly measured with parental reports and self-reports in research. Future studies, however, might need to combine the use of both parental/teacher reports and self-reports to ensure data triangulation or to develop new methodological tools that are less susceptible to bias.

## Conclusion

Of the 14 predictors included in our analysis, only RAN (measured in kindergarten) was uniquely predictive of adult reading fluency at age 23. RAN is considered a surface indicator of reading difficulties (a symptom rather than the cause), and no consensus has been reached on whether interventions aimed at training RAN can improve reading fluency (Norton & Wolf, 2012) because such intervention studies are rare and have reported mixed findings (Kirby et al., 2010; Stappen & Reybroeck, 2018). However, RAN can be effectively used for the early identification (even before school entry) of children at risk for adult reading difficulties, which can then facilitate timely interventions and support.

Importantly, the mechanisms behind resolving reading difficulties and the steepest trajectories of reading development remain largely unknown, and whether any environmental factors can be introduced into the education system to help children with low reading fluency get on a resolving track in their skill development is unclear. Our findings indicate the possibility of the intergenerational transmission of resolving trajectories, as those who had parents with resolving reading difficulties were more likely to achieve better reading fluency at age 23 than those whose parents had persistent difficulties. However, further research is needed to establish whether parents with resolving and persistent difficulties provide different learning environments for their children.

## Supplementary Material

Appendix - Growth curve article.docx

## Appendix

**Table A1.** Patterns of missingness in the predictor variables with largest missingness (>20%).

Predictor with missing values/missing percent	Children’s reading fluency whose values are present on the predictor N, Mean (SD)				Children’s reading fluency whose values are missing on the predictor N, Mean (SD)			
	Gr 2	Gr 3	Gr 8	Age 23	Gr 2	Gr 3	Gr 8	Age 23
Parental income, 22.5%	135, 5.32 (1.99)	150, 5.93 (1.74)	137, 8.79 (1.84)	102, 10.45 (2.07)	43, 4.63 (2.22)	50, 5.54 (2.22)	45, 8.36 (2.45)	34, 9.37 (2.45)
Task avoidance, ages 13–15, 34.6%	126, 5.25 (1.95)	133, 5.89 (1.91)	127, 8.60 (1.92)	100, 10.04 (2.22)	52, 4.90 (2.30)	67, 5.72 (1.80)	55, 8.89 (2.20)	36, 10.58 (2.15)
Leisure reading, ages,13–15, 44.5%	104, 5.14 (2.00)	112, 5.81 (1.91)	105, 8.57 (2.00)	85, 10.04 (2.23)	74, 5.17 (2.16)	88, 5.87 (1.84)	77, 8.85 (2.02)	51, 10.41 (2.18)

**Table A2.** Patterns of missingness in reading fluency at age 23.

Predictor with missing values/missing percent	Children’s reading fluency whose values are present on age 23 fluency N, Mean (SD)			Children’s reading fluency whose values are missing on age 23 fluency N, Mean (SD)		
	Gr 2	Gr 3	Gr 8	Gr 2	Gr 3	Gr 8
Age 23 reading fluency, 32.5%	128, 5.31 (2.03)	136, 5.99 (1.88)	132, 8.87 (1.98)	50, 4.75 (2.10)	64, 5.49 (1.82)	50, 8.22 (2.03)

Tables A1 and A2 do not reveal apparent patterns of systematic missingness but they do indicate that parents whose children had lower reading scores were slightly more likely not to report their income. In addition, children with lower reading scores were slightly more likely not to self-report their task avoidance at ages 13 and 15 and not to complete the reading fluency assessment at age 23.

**Table A3.** The fully standardized estimates of the additional models that included two growth specifications.

**Additional model 1 with two growth specifications: Cognitive predictors**		
Predictors	Intercept for the first growth model (Age 8), Estimate (SE)	Slope for the first growth model (Ages 8–9), Estimate (SE)
Verbal IQ, age 8	.107 (.076)	.009 (.093)
Performance IQ, age 8	.059 (.072)	−.013 (.089)
RAN, ages 5–6	**-.173** (.063)**	−.065 (.083)
Phonological awareness, age 5	−.013 (.098)	−.119 (.114)
Letter knowledge, age 5	.**419*** (.080)**	**-.219* (.090)**
Predictors	Intercept for the second growth model (Age 14), Estimate (SE)	Slope for the second growth model (Ages 14–23), Estimate (SE)
Verbal IQ, age 8	.043 (.084)	.215 (.120)
Performance IQ, age 8	.034 (.066)	−.016 (.105)
RAN, ages 5–6	**-.348*** (.070)**	.050 (.086)
Phonological awareness, age 5	−.117 (.094)	.203 (.123)
Letter knowledge, age 5	.**276** (.082)**	−.153 (.116)
**Additional model 2 with two growth specifications: Motivational predictors**		
Predictors	Intercept for the first growth model (Age 8), Estimate (SE)	Slope for the first growth model (Ages 8–9), Estimate (SE)
Reading motivation, ages 8–9	0.468*** (.068)	-.315*** (.088)
Task avoidance, age 9	−.076 (.081)	−.098 (.083)
Task avoidance, ages 13–15	.017 (.074)	−.108 (.079)
Leisure reading, ages 13–15	.093 (.086)	−.161 (.113)
Predictors	Intercept for the second growth model (Age 14), Estimate (SE)	Slope for the second growth model (Ages 14–23), Estimate (SE)
Reading motivation, ages 8–9	.186* (.094)	.176 (.108)
Task avoidance, age 9	−.124 (.085)	.019 (.090)
Task avoidance, ages 13–15	−.078 (.093)	−.063 (.105)
Leisure reading, ages 13–15	.001 (.092)	.099 (.141)
**Additional model 3 with two growth specifications: Parental predictors**		
Predictors	Intercept for the first growth model (Age 8), Estimate (SE)	Slope for the first growth model (Ages 8–9), Estimate (SE)
Parental education	.152 (.087)	-.152* (.075)
Parental income	−.022 (.113)	−.076 (.101)
Parental dyslexia	-.269*** (.066)	.149* (.073)
Shared reading, ages 4–6	.065 (.071)	−.059 (.088)
Teaching letters, 4	.111 (.075)	−.049 (.093)
Predictors	Intercept for the second growth model (Age 14), Estimate (SE)	Slope for the second growth model (Ages 14–23), Estimate (SE)
Parental education	.109 (.087)	.026 (.095)
Parental income	−.055 (.119)	−.001 (.094)
Parental dyslexia	-.215** (.069)	.005 (.082)
Shared reading, ages 4–6	−.042 (.072)	.027 (.077)
Teaching letters, 4	.108 (.073)	.267*** (.071)
**Additional model 4 with two growth specifications: All significant predictors**		
Predictors	Intercept for the first growth model (Age 8), Estimate (SE)	Slope for the first growth model (Ages 8–9), Estimate (SE)
Parental dyslexia	-.169** (.057)	.138* (.070)
RAN, ages 5–6	-.188** (.059)	−.077 (.083)
Letter knowledge, age 5	.347*** (.063)	-.217** (.081)
Reading motivation, ages 8–9	.385*** (.055)	-.178* (.078)
Teaching letters, age 4	−.046 (.062)	.024 (.095)
Predictors	Intercept for the second growth model (Age 14), Estimate (SE)	Slope for the second growth model (Ages 14–23), Estimate (SE)
Parental dyslexia	−.118 (.067)	−.022 (.086)
RAN, ages 5–6	-.346*** (.066)	.020 (.085)
Letter knowledge, age 5	.163* (.075)	−.078 (.095)
Reading motivation, ages 8–9	.163* (.076)	.089 (.100)
Teaching letters, age 4	.003 (.068)	.290*** (.076)
**Additional model 5 with two growth specifications: Dummy predictors of parental dyslexia**		
Predictor	Intercept for the first growth model (Age 8), Estimate (SE)	Slope for the first growth model (Ages 8–9), Estimate (SE)
Persistent parental difficulties	-.323*** (.067)	.166* (.077)
Resolving parental difficulties	-.159*(.078)	.156* (.070)
	Intercept for the second growth model (Age 14), Estimate (SE)	Slope for the second growth model (Ages 14–23), Estimate (SE)
Persistent parental difficulties	-.300***(.067)	−.030 (.097)
Resolving parental difficulties	−.053 (.086)	−.029 (.070)

**p* < .05, ***p* < .01, ****p* < .001. All models in this table are saturated and for this reason fit statistics are not reported.
