# Supplementary material for: Predicting Reading Fluency Growth from Grade 2 to Age 23 with Parental and Child Factors
Source: Sci Stud Read. 2024 Apr 27;28(5):485–509. doi: 10.1080/10888438.2024.2346323 (PMC11346386; doi:10.1080/10888438.2024.2346323)
Supplement: Appendix - Growth curve article.docx [file HSSR_A_2346323_SM5420.docx]

**Appendix**

**Table 1**

Patterns of missingness in the predictor variables with largest missingness (>20%)

| Predictor with missing values/ missing percent | Children’s reading fluency whose values are present on the predictor  N, Mean (SD) | | | | Children’s reading fluency whose values are missing on the predictor  N, Mean (SD) | | | |
| --- | --- | --- | --- | --- | --- | --- | --- | --- |
|  | Gr 2 | Gr 3 | Gr 8 | Age 23 | Gr 2 | Gr 3 | Gr 8 | Age 23 |
| Parental income, 22.5% | 135, 5.32 (1.99) | 150, 5.93 (1.74) | 137, 8.79 (1.84) | 102, 10.45 (2.07) | 43, 4.63 (2.22) | 50, 5.54 (2.22) | 45, 8.36 (2.45) | 34, 9.37 (2.45) |
| Task avoidance, ages 13-15, 34.6% | 126, 5.25 (1.95) | 133, 5.89 (1.91) | 127, 8.60 (1.92) | 100, 10.04 (2.22) | 52, 4.90 (2.30) | 67, 5.72 (1.80) | 55, 8.89 (2.20) | 36, 10.58 (2.15) |
| Leisure reading, ages,13-15, 44.5% | 104, 5.14 (2.00) | 112, 5.81 (1.91) | 105, 8.57 (2.00) | 85, 10.04 (2.23) | 74, 5.17 (2.16) | 88, 5.87 (1.84) | 77, 8.85 (2.02) | 51, 10.41 (2.18) |

**Table 2**

Patterns of missingness in reading fluency at age 23

| Predictor with missing values/ missing percent | Children’s reading fluency whose values are present on age 23 fluency  N, Mean (SD) | | | Children’s reading fluency whose values are missing on age 23 fluency  N, Mean (SD) | | |
| --- | --- | --- | --- | --- | --- | --- |
|  | Gr 2 | Gr 3 | Gr 8 | Gr 2 | Gr 3 | Gr 8 |
| Age 23 reading fluency, 32.5% | 128,  5.31 (2.03) | 136,  5.99 (1.88) | 132,  8.87 (1.98) | 50,  4.75 (2.10) | 64,  5.49 (1.82) | 50,  8.22 (2.03) |

Tables 1 and 2 do not reveal apparent patterns of systematic missingness but they do indicate that parents whose children had lower reading scores were slightly more likely not to report their income. In addition, children with lower reading scores were slightly more likely not to self-report their task avoidance at ages 13 and 15 and not to complete the reading fluency assessment at age 23.

**Table 3**

The fully standardized estimates of the additional models that included two growth specifications

| **Additional model 1 with two growth specifications: Cognitive predictors** | | |
| --- | --- | --- |
| Predictors | Intercept for the first growth model (Age 8), Estimate (SE) | Slope for the first growth model (Ages 8-9), Estimate (SE) |
| Verbal IQ, age 8 | .107 (.076) | .009 (.093) |
| Performance IQ, age 8 | .059 (.072) | -.013 (.089) |
| RAN, ages 5-6 | **-.173** (.063)** | -.065 (.083) |
| Phonological awareness, age 5 | -.013 (.098) | -.119 (.114) |
| Letter knowledge, age 5 | **.419*** (.080)** | **-.219* (.090)** |
| Predictors | Intercept for the second growth model (Age 14), Estimate (SE) | Slope for the second growth model (Ages 14-23), Estimate (SE) |
| Verbal IQ, age 8 | .043 (.084) | .215 (.120) |
| Performance IQ, age 8 | .034 (.066) | -.016 (.105) |
| RAN, ages 5-6 | **-.348*** (.070)** | .050 (.086) |
| Phonological awareness, age 5 | -.117 (.094) | .203 (.123) |
| Letter knowledge, age 5 | **.276** (.082)** | -.153 (.116) |
| **Additional model 2 with two growth specifications: Motivational predictors** | | |
| Predictors | Intercept for the first growth model (Age 8), Estimate (SE) | Slope for the first growth model (Ages 8-9), Estimate (SE) |
| Reading motivation, ages 8-9 | **0.468*** (.068)** | **-.315*** (.088)** |
| Task avoidance, age 9 | -.076 (.081) | -.098 (.083) |
| Task avoidance, ages 13-15 | .017 (.074) | -.108 (.079) |
| Leisure reading, ages 13-15 | .093 (.086) | -.161 (.113) |
| Predictors | Intercept for the second growth model (Age 14), Estimate (SE) | Slope for the second growth model (Ages 14-23), Estimate (SE) |
| Reading motivation, ages 8-9 | **.186* (.094)** | .176 (.108) |
| Task avoidance, age 9 | -.124 (.085) | .019 (.090) |
| Task avoidance, ages 13-15 | -.078 (.093) | -.063 (.105) |
| Leisure reading, ages 13-15 | .001 (.092) | .099 (.141) |
| **Additional model 3 with two growth specifications: Parental predictors** | | |
| Predictors | Intercept for the first growth model (Age 8), Estimate (SE) | Slope for the first growth model (Ages 8-9), Estimate (SE) |
| Parental education | .152 (.087) | **-.152* (.075)** |
| Parental income | -.022 (.113) | -.076 (.101) |
| Parental dyslexia | **-.269*** (.066)** | **.149* (.073)** |
| Shared reading, ages 4-6 | .065 (.071) | -.059 (.088) |
| Teaching letters, 4 | .111 (.075) | -.049 (.093) |
| Predictors | Intercept for the second growth model (Age 14), Estimate (SE) | Slope for the second growth model (Ages 14-23), Estimate (SE) |
| Parental education | .109 (.087) | .026 (.095) |
| Parental income | -.055 (.119) | -.001 (.094) |
| Parental dyslexia | **-.215** (.069)** | .005 (.082) |
| Shared reading, ages 4-6 | -.042 (.072) | .027 (.077) |
| Teaching letters, 4 | .108 (.073) | **.267***** **(.071)** |
| **Additional model 4 with two growth specifications: All significant predictors** | | |
| Predictors | Intercept for the first growth model (Age 8), Estimate (SE) | Slope for the first growth model (Ages 8-9), Estimate (SE) |
| Parental dyslexia | **-.169** (.057)** | **.138* (.070)** |
| RAN, ages 5-6 | **-.188** (.059)** | -.077 (.083) |
| Letter knowledge, age 5 | **.347*** (.063)** | **-.217** (.081)** |
| Reading motivation, ages 8-9 | **.385*** (.055)** | **-.178* (.078)** |
| Teaching letters, age 4 | -.046 (.062) | .024 (.095) |
| Predictors | Intercept for the second growth model (Age 14), Estimate (SE) | Slope for the second growth model (Ages 14-23), Estimate (SE) |
| Parental dyslexia | -.118 (.067) | -.022 (.086) |
| RAN, ages 5-6 | **-.346*** (.066)** | .020 (.085) |
| Letter knowledge, age 5 | **.163* (.075)** | -.078 (.095) |
| Reading motivation, ages 8-9 | **.163* (.076)** | .089 (.100) |
| Teaching letters, age 4 | .003 (.068) | **.290*** (.076)** |
| **Additional model 5 with two growth specifications: Dummy predictors of parental dyslexia** | | |
| Predictor | Intercept for the first growth model (Age 8), Estimate (SE) | Slope for the first growth model (Ages 8-9), Estimate (SE) |
| Persistent parental difficulties | **-.323*** (.067)** | **.166* (.077)** |
| Resolving parental difficulties | **-.159*(.078)** | **.156* (.070)** |
|  | Intercept for the second growth model (Age 14), Estimate (SE) | Slope for the second growth model (Ages 14-23), Estimate (SE) |
| Persistent parental difficulties | **-.300***(.067)** | -.030 (.097) |
| Resolving parental difficulties | -.053 (.086) | -.029 (.070) |

*Note.* * *p*<.05, ** *p*<.01, *** *p*<.001. All models in this table are saturated and for this reason fit statistics are not reported.
